# Perceived patient burden and acceptability of whole body MRI for staging lung and colorectal cancer; comparison with standard staging investigations

**DOI:** 10.1259/bjr.20170731

**Published:** 2018-03-20

**Authors:** Ruth EC Evans, Stuart A Taylor, Sandra Beare, Steve Halligan, Alison Morton, Alf Oliver, Andrea Rockall, Anne Miles

**Affiliations:** 1 Deparment of Psychological Sciences, Birkbeck, University of London, London, UK; 2 Division of Medicine, Centre for Medical Imaging, University College London, London, UK; 3 Cancer Research UK and UCL Cancer Trials Centre, London, UK; 4 C/O National Cancer Research Institute, Angel Building, London, UK; 5 Department of Surgery and Cancer, Imperial College London, Kensington, London, UK; 6 Department of Radiology, Royal Marsden NHS Foundation Hospital Trust, London, UK; 7 Streamline Investigators listed in the collaborators section

## Abstract

**Objective:**

To evaluate perceived patient burden and acceptability of whole body MRI (WB-MRI) compared to standard staging investigations, and identify predictors of reduced tolerance.

**Methods:**

Patients recruited to multicentre trials comparing WB-MRI with standard staging scans for lung and colorectal cancer were invited to complete two questionnaires: a baseline questionnaire at recruitment, measuring demographics, comorbidities, and distress; and a follow-up questionnaire after staging, measuring recovery time, comparative acceptability/satisfaction between WB-MRI and CT (colorectal cancer) and PET-CT (lung cancer), and perceived scan burden (scored 1, low; 7, high).

**Results:**

115 patients (median age 66.3 years; 67 males) completed follow up and 103 baseline questionnaires. 69 (63.9%) reported “immediate” recovery from WB-MRI and 73 (65.2%) judged it “very acceptable”. Perceived WB-MRI burden was greater than for CT (*p *< 0.001) and PET-CT (*p *< 0.001). High distress and comorbidities were associated with greater WB-MRI burden in adjusted analyses, with deprivation only approaching significance (adjusted regression β = 0.223, *p *= 0.025; β = 0.191, *p *= 0.048; β = −0.186, *p *= 0.059 respectively). Age (*p *= 0.535), gender (*p *= 0.389), ethnicity (*p *= 0.081) and cancer type (*p *= 0.201) were not predictive of WB-MRI burden.

**Conclusion:**

WB-MRI is marginally less acceptable and more burdensome than standard scans, particularly for patients with pre-existing distress and comorbidities.

**Advances in knowledge:**

This research shows that WB-MRI scan burden, although low, is higher than for current staging modalities among patients with suspected colorectal or lung cancer. Psychological and physical comorbidities adversely impact on patient experience of WB-MRI. Patients with high distress or comorbid illness may need additional support to undergo a WB-MRI.

## Introduction

Patients diagnosed with cancer must be staged accurately prior to treatment decisions. In particular, it is imperative to detect metastatic disease, as this impacts considerably on therapeutic approach. Standard staging pathways are often complex, time consuming and involve several different imaging modalities, potentially adding to physical and psychological burden of patients with known or suspected cancer.^1^

Recent data suggest whole body MRI (WB-MRI) has potential as an “all-in-one” staging investigation that at least matches and possibly betters the accuracy of conventional investigations for detecting metastatic disease.^2, 3^ One critical but often neglected aspect influencing adoption of any new technology is patient experience. Low patient acceptability reduces adherence, which diminishes diagnostic impact, even when superior to existing tests. Uptake of bowel cancer screening colonoscopy is an example where perceived test burden impacts directly to reduce participation.^4^

WB-MRI has several attributes that can impact negatively on patient experience. Although protocols are dependent upon the underlying disease process, the scan acquisition time for cancer staging is typically around 45–60 min, and considerably longer than CT or even PET-CT, with image acquisitions taking seconds or minutes respectively (although patient experience will be influenced by the total examination time, rather than just time taken for image acquisition). Moreover, MRI scanners are noisy and require full body and head immersion inside a relatively narrow “tube”, often necessitating closely applied receiver coils that restrict movement. Existing data show that 5–30% of patients experience distress both in anticipation of MRI, and during the scan itself.^5–7^ Severe claustrophobia terminates scanning in 1–15%,^8^ and even if the patient completes the scan, distress precipitates motion artefacts that degrade image quality and impair diagnostic accuracy.^9^ Furthermore, post-scan anxiety^6^ can engender MRI fear or phobia.^10^

Quantifying patient “distress” around diagnostic imaging is complex and has been expressed as procedural “burden”, a composite variable based on rating the level of physical and psychological discomfort related to scanning. Shortman et al^11^ found the perceived burden of PET-MRI was greater than PET-CT; burden was related to scan preference with an overall preference for PET-CT. A recent qualitative interview study reported that WB-MRI was perceived by some as more challenging than PET-CT and CT.^12^ To date, predictors of increased patient burden before or during WB-MRI have received little attention. Such knowledge may identify those who require additional psychological support in advance or physical interventions such as sedation in order to complete scanning.^13^

The purpose of this study was to evaluate the perceived patient burden and acceptability of WB-MRI compared to standard staging investigations, and to identify predictors of reduced patient tolerance.

## Methods and materials

### Participants

Patients recruited prospectively to two ongoing clinical trials, comparing the diagnostic accuracy and cost-effectiveness of WB-MRI with standard tests for staging colorectal and lung cancer, were invited to participate in the current study. Patients were eligible for the main trials if they were recently diagnosed or highly suspected of colorectal (Streamline C) or non-small cell lung cancer (Streamline L), such that they were referred for staging investigations. Written consent was obtained for participation in the current study. As part of the trial protocol, patients underwent WB-MRI staging in addition to all standard staging investigations such as CT and PET-CT. The full trial protocol details have been previously reported.^14^ The WB-MRI required intravenous cannulation for the administration of gadolinium. Full ethical permission was given by Camden and Islington National Research Ethics Service (NRES) on 03/10/2012, project numbers: 12/LO/1176 (Streamline C) and 12/LO/1177 (Streamline L).

Between March 2013 and July 2015, 392 consecutive patients recruited to the main trials were given the option to participate in either an interview study (reported elsewhere)^12^ or the current questionnaire study as part of the informed consent process for the main trials. 350 (89.3%) consented. The interview study investigated patients’ experiences of staging investigations.

Initially, patients (*n* = 91) were recruited to the interview study, previously reported.^12^ Thereafter, patients were recruited exclusively to the questionnaire study presented here. None of the patients who took part in the present study took part in the prior interview study.

**Figure 1.**
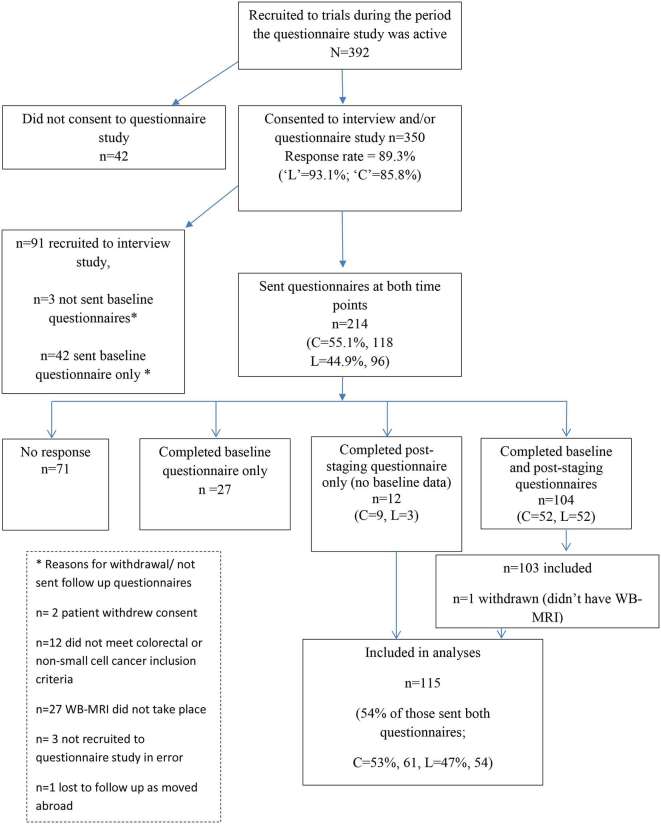
Flow diagram of participants through the study (March 2013–July 2015).

The full recruitment pathway and reasons for exclusion is presented in Figure 1. A total of 115 patients completing the follow up questionnaires (see below) were included in the analyses.

### Procedures

Patients were asked to complete two questionnaires. The first (baseline) questionnaire was mailed to patients within 2 days of being registered for the Streamline trials, completed around the time patients were undergoing their staging investigations, and returned using a stamped addressed reply envelope. A second “post-staging” questionnaire was posted 1 month after the baseline questionnaire was administered and was completed after all staging investigations were completed. Patients were paid £20 for participation, which was continued until a minimum of 100 patients had returned both questionnaires (50 for Streamline L and 50 for Streamline C)—see power calculation below.

### Questionnaire content

The following data were collected in the baseline questionnaire:

- Emotional distress: the 12-item General Health Questionnaire (GHQ-12)^15^ was used to assess psychological distress. An example item is, *“In the last three months have you….been able to concentrate on whatever you’re doing”*. Using the GHQ-12 binary coding method (0,0,1,1), a mean sum score (if at least 50% of items were answered) was created ranging from 0 to 12. A score of 4 or higher is considered indicative of significant distress levels.^16^
- Comorbidity: patients were asked about their current and recent physical health and mental well-being. Patients were asked to report (“yes” or “no”) whether they had any of the following diseases: heart or vascular disease, diabetes, epilepsy, stroke, arthritis, asthma, mental or emotional disorder. There was also an option to provide details of other illness. A response of “yes” to any illness was coded and a dichotomous “comorbidity” variable was created, whereby the presence of one or more comorbid illness was reported: either yes or no. The presence of a mental or emotional disorder was excluded as this was captured in the GHQ-12. Self-report measures of comorbidity have been shown to be valid^17, 18^ and offer a more cost-effective method of data collection than medical record-based measures.
- Demographics: patients were asked their age, gender and ethnicity. Missing demographic data on age and gender as well as zip code data were supplied via the central trial database (with patient consent). Zip code data were used to calculate an area based deprivation score for each individual using the 2010 Index of Multiple Deprivation scale,^19 ^categorised into quintiles from 1 (highest levels of deprivation) to 5 (lowest).

Part of the follow-up questionnaire asked patients about their comparative experience of WB-MRI and staging CT chest, abdomen and pelvis (standard scan) if recruited to Streamline C, or to PET-CT (standard scan) if recruited to Streamline L. The following data were captured.

- Scan recovery, satisfaction and acceptability: patients rated their post-scan recovery on a 9-point scale ranging from “immediate” to “a week”. Data were collapsed into three categories “immediate”, “up to 30 min” and “over 30 min” for analysis. Patients also rated how satisfied they were with the information received before scanning, communication and departmental facilities, as well as the overall acceptability of scans, on a scale of 1 (very dissatisfied/ not at all acceptable) to 4 (very satisfied/ very acceptable).
- Scan burden was quantified using a questionnaire adapted from one previously used to assess acceptability of colonoscopy^20, 21^ (Supplementary material 1a, Supplementary material available online). Patients completed the 26-item scale for both WB-MRI and standard scans, describing their experiences by ticking agreement on a 1–7 Likert scale, where 1 and 7 were anchored to bipolar statements related to scan discomfort (13 items), worry (6 items), and satisfaction (7 items). An example discomfort item was 1= “not claustrophobic” to 7= “claustrophobic”. Subscores for discomfort, worry and satisfaction scales were computed from the mean of completed items (if less than 50% of items were completed, the response was coded as missing). A total score “scan burden” was computed by taking the mean of discomfort, worry and reverse scored satisfaction subscales with higher scores equating to greater scan burden.

### Power calculation

Power (G*Power-v. 3)^22^ was based on rejecting the null hypothesis that there was no significant difference in perceived burden of WB-MRI when compared to standard staging (related *t*-test). Assuming a medium effect size (d = 0.5), α of 0.05 and 95% power,^23^ a minimum number of 90 patients were required across the two study cohorts (45 in Streamline C and 45 in Streamline L). An effect size of 0.5 is considered the minimal important difference in quality of life measures,^24^ where minimal important difference is defined as the smallest difference that patients view as important (beneficial or harmful), and would result in a doctor considering a change in the patient’s management.^25^

### Statistical analysis

Analysis was performed using SPSS v. 22. Differences in demographic and psychological characteristics between Streamline L and Streamline C cohorts were assessed using the Mann–Whitney *U* test, and *X*^*2*^ or Fisher’s Exact tests (if 20% or more of the cells in the contingency table had expected counts of less than 5) as appropriate. Related samples Wilcoxon sign tests were used to assess differences between WB-MRI * vs * CT/PET-CT in terms of scan recovery time, scan acceptability, and satisfaction with scan-related information, facilities, communication and scan burden. Linear regression tested the predictive value for WB-MRI scan burden of data collected in the baseline questionnaire. Individual predictors were entered in unadjusted analyses and those items achieving statistical significance were then entered into a multivariate analysis. Statistical significance was assigned at the 5% level, two-tailed.

## Results

Of the 350 patients agreeing to participate in the questionnaire or separate interview study, rates of consent were significantly higher among patients recruited to Streamline L compared to those recruited to Streamline C; (93.1* vs *85.8%; *X*^2^ = 5.451, df = 1; *p* = 0.020, Figure 1). There were no differences in basic demographics between those who consented compared to those who did not (Supplementary material 1b).

 In total, 214 patients were sent both questionnaires of whom 99 were excluded leaving 115 for analysis. Reasons for exclusion were non-response (*n* = 71), returned baseline questionnaire only (*n* = 27), and trial withdrawal (*n* = 1) (Figure 1). Patients with lower levels of deprivation were more likely to return the post-staging questionnaire (linear *X*^2^ = 7.113, df = 1; *p* = 0.008). There were no differences in sex (*p* = 0.059), age (*p* = 0.676) or cancer type (*X*^2^ = 0.442; df = 1; *p* = 0.506), between those who did, and did not return the post staging questionnaire (Supplementary material 1c).

Full demographics of the 115 patients are shown in Table 1. Overall, 103 patients (median age 66; 58 males) completed both questionnaires and 12 (median age 60; 9 males) completed the post-staging questionnaire only. 61 patients were recruited to Streamline C and 54 to Streamline L. Female patients recruited to Streamline C (*n* = 24) were significantly younger than those recruited to Streamline L (*n* = 24) (median age 60* vs *73 years; *p* = 0.003), with no significant age difference between males (66 years, *n* = 37 *vs* 66 years, *n* = 30, respectively; *p* = 0.480).

**Table 1.** Demographic and psychological characteristics of participants who completed the post-staging questionnaire

	Overall	Patient cohort		Differences between patient cohorts
*N* = 115	L^*a*^ *n* = 54	C^*b*^ *n* = 61
Demographic characteristics				
Age^*c*^ [median in years (range)]	66.3 (31–89)	69.7 (50–89)	64.2 (31–85)	Mann–Whitney *U* test *p* = 0.010
Male gender^*c*^	58.3 (67)	55.6 (30)	60.7 (37)	*X^2^* = 0.306; df = 1; *p* = 0.580
White ethnicity^*d*^	91.8 (90)	93.8 (45)	90.0 (45)	Fisher’s Exact; *p* = 0.715
IMD deprivation^*c*^				
1 (highest)	23.5 (27)	25.9 (14)	21.3 (13)	*X^2^ = *0.3875; df = 4; *p* = 0.423
2	24.3 (28)	27.8 (15)	21.3 (13)
3	21.7 (25)	24.1 (13)	19.7 (12)
4	17.4 (20)	14.8 (8)	19.7 (12)
5 (lowest)	13.0 (15)	7.4 (4)	18.0 (11)
Physical and emotional well-being				
Comorbidity (at least one comorbid illness reported)^*d*^	53.4 (55)	66.7 (34)	40.4 (21)	*X^2^* = 7.147; df = 1; *p* = 0.008
Emotional distress^*d*^				
(GHQ-12 score of 4 or higher)	41.6 (42)	47.1 (24)	36.0 (18)	*X^2^* = 1.271; df = 1; *p* = 0.260

GHQ-12, 12-Item General Health Questionnaire; IMD, Index of Multiple Deprivation.

Numbers are percent (n) unless otherwise specified.

a Non-small cell lung cancer.

b Colorectal cancer % is valid percent where there is missing data.

c No missing data.

d Missing data greater than 5%.

Patients recruited to Streamline L were significantly more likely to report additional comorbidity than those recruited to Streamline C (66.7* vs *40.4%, *p* = 0.008) with no significant differences for the presence of baseline psychological distress between the two trial cohorts (Table 1).

### Post-scan patient recovery and scan acceptability

Patients’ responses to scan recovery time and overall acceptability are summarised in Table 2. There were no significant differences in recovery time after WB-MRI compared to CT/PET CT, with 63.9% of patients who completed this item (*n* = 69) reporting “immediate” recovery following WB-MRI compared with 65.1% following CT/PET-CT (Table 2). However, scan acceptability ratings were significantly lower for WB-MRI compared to both CT and PET-CT. Patients’ satisfaction with information before the scan and facilities, together with communication during the scan and were all high and not significantly different between WB-MRI and either CT or PET-CT (Table 2).

**Table 2.** Comparative experience of WB-MRI * vs * CT/PET-CT

	Overall	Lung (L)*^a^*	Colorectal (C)*^b^*	Group differences (Wilcoxon sign test)
Recovery time				
WB-MRI^*c*^				*p* = 0.465*^*d*^p *= 0.735^*e *^
Immediate	63.9 (69)	61.5 (32)^**d**^	66.1 (37)*^*e *^*
Up to 30 min	25.9 (28)	23.1 (12)	28.6 (16)
Over 30 min	10.2 (11)	15.4 (8)	5.4 (3)
CT/PET-CT^*c*^			
Immediate	65.1 (69)	58.8 (30)^*d*^	70.9 (39)^*e *^
Up to 30 min	21.7 (23)	23.5 (12)	20.0 (11)
Over 30 min	13.2 (14)	17.6 (9)	9.1 (5)
Acceptability				
WB-MRI^*c*^				*p* = 0.035﻿^*d*^*p* = 0.005﻿^*e *^
Very	65.2 (73)	64.8 (35)^*d*^	65.5 (38)^*e *^
Fairly	30.4 (34)	29.6 (16)	31.0 (18)
Slightly	3.6 (4)	3.7 (2)	3.4 (2)
Not at all	0.9 (1)	1.9 (1)	0.0 (0)
CT/PET-CT^*c*^			
Very	77.8 (84)	75.0 (39)^*d*^	80.4 (45)^*e *^
Fairly	21.3 (23)	23.1 (12)	19.6 (11)
Slightly	0.0 (0)	0.0 (0)	0.0 (0)
Not at all	0.9 (1)	1.9 (1)	0.0 (0)
Satisfied with information received before scan				
WB-MRI^*c*^				*p* = 0.169^*d*^*p* = 0.071^*e *^
Very satisfied	55.6 (60)	51.9 (27)^*d*^	58.9 (33)^*e *^
Satisfied	37.0 (40)	40.4 (21)	33.9 (19)
Dissatisfied	3.7 (4)	5.8 (3)	1.8 (1)
Very dissatisfied	3.7 (4)	1.9 (1)	5.4 (3)
CT/PET-CT^*c*^			
Very satisfied	57.5 (61)	49.0 (25)^*d*^	65.5 (36)^*e *^
Satisfied	34.9 (37)	37.3 (19)	32.7 (18)
Dissatisfied	0.9 (1)	2.0 (1)	0 (0)
Very dissatisfied	6.6 (7)	11.8 (6)	1.8 (1)
Satisfied with communication during scan				
WB-MRI^*c*^				*p* = 0.637*^*d*^* *p* = 0.059^*e *^
Very satisfied	56.1 (60)	57.7 (30)^*d*^	54.5 (30)^*e *^
Satisfied	39.3 (42)	34.6 (18)	43.6 (24)
Dissatisfied	2.8 (3)	5.8 (3)	0 (0)
Very dissatisfied	1.9 (2)	1.9 (1)	1.8 (1)
CT/PET-CT^*c*^			
Very satisfied	64.2 (68)	62.7 (32)^*d*^	65.5 (36)^*e *^
Satisfied	32.1 (34)	31.4 (16)	32.7 (18)
Dissatisfied	1.9 (2)	3.9 (2)	0 (0)
Very dissatisfied	1.9 (2)	2.0 (1)	1.8 (1)
Satisfaction with facilities				
WB-MRI^*c*^				*p* = 0.225^*d*^*p* = 0.480^*e *^
Very satisfied	45.8 (49)	49.0 (25)^*d*^	42.9 (24)^*e *^
Satisfied	45.8 (49)	43.1 (22)	48.2 (27)
Dissatisfied	4.7 (5)	2.0 (1)	7.1 (4)
Very dissatisfied	3.7 (4)	5.9 (3)	1.8 (1)
CT/PET-CT^*c*^			
Very satisfied	54.7 (58)	62.7 (32)^*d*^	47.3 (26)^*e *^
Satisfied	38.7 (41)	33.3 (17)	43.6 (24)
Dissatisfied	4.7 (5)	2.0 (1)	7.3 (4)
Very dissatisfied	1.9 (2)	2.0 (1)	1.8 (1)

WB-MRI, whole body MRI.

Numbers are percent (n).

a Non-small cell lung cancer, WB-MRI *vs* PET-CT.

b Colorectal cancer, WB-MRI *vs* CT.

c Missing data greater than 5%. % is valid percent.

d Comparison between WB-MRI and PET-CT (Lung).

e Comparison between WB-MRI and CT (Colorectal).

### Scan burden

In general, patients tolerated all the imaging modalities well and reported low levels of scan burden. Mean ratings for scan discomfort and worry ranged from 1.63 to 2.65, where 7 represents maximum discomfort or worry. Mean satisfaction scores ranged from 6.25 to 6.53, where 7 represents maximum satisfaction.

However, mean burden scores for WB-MRI were significantly greater than those of PET-CT and CT (Table 3). The higher burden of WB-MRI was mainly due to items related to “discomfort”, although there were also significant differences in relation to “satisfaction”. Questionnaire items related to “worry” were only less favourable for WB-MRI in comparison to CT, and did not differ for WB-MRI in comparison to PET-CT. Specific items within the discomfort subscale particularly relevant to WB-MRI, showed WB-MRI conferred significantly greater feelings of claustrophobia than both CT (means scores 2.81* vs *1.51; *p* < 0.001) and PET-CT (mean scores 3.04* vs *1.98; *p* < 0.001); greater burden from scan-related noise compared with both CT (means 2.84* vs *1.73; *p* < 0.001) and PET-CT (2.85* vs *1.63; *p* < 0.001). In general, the intravenous injections required for each of the three scan types resulted in low levels of discomfort which did not differ between scan type (WB-MRI * vs * CT: 1.59* vs *1.56, *p* = 0.637; WB-MRI * vs * PET-CT: 1.86* vs *1.73, *p* = 0.225).

**Table 3.** Comparative scan burden (WB-MRI * vs * CT/PET-CT)

	Overall	L^*a*^ cohort	C^*b*^ cohort	Group differences using Wilcoxon signed-rank test
Total patient burden (scores 1–7)				
WB-MRI^*c *^	2.21 (1.1)	2.33*^*d*^* (0.94)	2.09^*e*^ (1.18)	*p* < 0.001*^*d*^*
CT/PET-CT^*c*^	1.87 (0.98)	2.05^*d*^ (0.82)	1.70^*e*^ (1.1)	*p* < 0.001*^*e*^*
Discomfort sub-scale (1–7)				
WB-MRI^*c*^	2.51 (1.26)	2.65^*d*^ (1.14)	2.30^*e*^ (1.22)	*p* < 0.001*^*d*^*
CT/PET-CT^*c*^	1.83 (1.05)	2.04*^*d*^* (.90)	1.63^*e*^ (1.15)	*p* < 0.001^*e*^
Worry subscale (1–7)				
WB-MRI^*c*^	2.47 (1.32)	2.62*^*d*^* (1.15)	2.23^*e*^ (1.31)	*p* = 0.208*^*d*^*
CT/PET-CT^*c*^	2.24 (1.23)	2.52^*d*^ (1.15)	2.00^*e*^ (1.28)	*p* = 0.041*^*e*^*
Satisfaction subscale (1–7)				
WB-MRI^*c*^	6.25 (1.06)	6.27*^*d*^* (0.85)	6.26^*e*^ (1.23)	*p* = 0.036﻿^*d*^
CT/PET-CT^*c*^	6.49^*f*^ (0.89)	6.43*^*d*^* (0.76)	6.53^*e*^ (1.01)	*p* < 0.001﻿^*e*^

WB-MRI, whole body MRI.

Numbers are mean (SD).

a Non-small cell lung cancer, WB-MRI *vs* PET-CT.

b Colorectal cancer, WB-MRI *vs* CT.

c Missing data greater than 5%.

d Comparison between WB-MRI and PET-CT (Lung).

e Comparison between WB-MRI and CT (Colorectal).

WB-MRI burden was not rated differently between those recruited to Streamline C or Streamline L cohorts (see below). In contrast, patients recruited to Streamline L reported significantly more worry and discomfort during PET-CT compared to the equivalent ratings for CT by those recruited to Streamline C; (worry 2.52* vs *2.00; *p* < 0.001; discomfort 2.04* vs *1.63: *p* < 0.001).

### Predictors of WB-MRI scan burden

The regression analysis for predictors of WB-MRI scan burden showed that the presence of comorbidity, psychological distress and deprivation were significant predictors in unadjusted analysis (β = 0.242, *p* = 0.015, β = 0.305, *p* = 0.002 and β = −0.265, *p* = 0.005 respectively), with age, gender, and cancer type non-significant predictors and ethnicity approaching significance (β = 0.059, *p* = 0.535; β = 0.083, *p* = 0.389; β = −0.122, *p* = 0.201; β = −0.179, *p* = 0.081). In the adjusted analyses, only psychological distress and presence of comorbidities remained significantly predictive (β = 0.223; *p* = 0.025; β = 0.191, *p* = 0.048) with deprivation approaching significance (β = −0.186, *p* = 0.059).

## Discussion

As data supporting WB-MRI for cancer staging accumulates^2, 3^ and the technology enters clinical practice, it is important to understand patient experience and overall acceptability. Cancer patients are vulnerable and may already be suffering significant distress^1, 26^ which may impact on the acceptability of potentially unpleasant staging investigations.

We investigated patient experience and overall acceptability of WB-MRI compared to standard PET-CT and CT in two cohorts of patients recently diagnosed or highly suspected of lung or colorectal cancer. While standard scans can distress patients,^27, 28^ we hypothesised that patients would find WB-MRI less acceptable given its attributes. This hypothesis was informed by related qualitative work that indicated some (but not all) patients found the scan a challenge and comparatively more so than CT and PET-CT scans.^12^

In reality, our data show that, in general, patients tolerate WB-MRI well; absolute discomfort and worry were low, and satisfaction was high. However, the burden of WB-MRI was significantly greater than for both PET-CT and CT. This differential was particularly apparent when compared to CT, the standard first-line staging investigation for patients with colorectal cancer. We also found evidence that PET-CT burden was greater than for CT, particularly for items pertaining to discomfort and worry, although, as noted below, the higher prevalence of comorbidities in the lung cancer patient cohort may have influenced their tolerance of PET-CT.

Although our findings are perhaps intuitive given the known attributes of the tests, they are actually at odds with the findings of Adams et al^29^ who compared WB-MRI with CT in patients undergoing lymphoma staging. Adams found that patients found WB-MRI more “friendly”, less unpleasant, and less “worrisome” than CT, attributing the relative negative evaluation of CT to more invasive preparation—patients had an intravenous line placed and consumed oral contrast. In our study, mean patient age (65 years) was considerably higher than the 50 years reported by Adams et al furthermore, the Streamline trial WB-MRI protocols required IV gadolinium, which may also help explain discrepant findings.

We investigated factors that might predict worsened scan experience. As would perhaps be expected in a cohort of patients undergoing investigations for suspected or newly diagnosed cancer, a significant proportion reported high level of baseline distress, and this distress was associated with subsequent higher WB-MRI burden. Furthermore, patients with additional comorbidity experienced greater burden. A recent review suggests that comorbidities can reduce cancer survival and comorbidity is associated with receiving suboptimal treatment.^30^ Our data suggest comorbidity influences the tolerability of WB-MRI, which may impact on scan quality and diagnostic accuracy. Further exploration of how comorbidity influences patients' experience of cancer staging and treatment is, therefore, important to maximise survival. High deprivation was associated with increased WB-MRI burden in the unadjusted analysis. Deprivation is associated with higher cancer incidence and mortality, particularly for lung cancer,^31^ in addition to decreased engagement with cancer screening programmes.^32^ Further work to understand how deprivation influences perceived burden is important to improve experience and engagement.

Our study does have limitations. Patients recruited to the Streamline trials volunteered to take part in our questionnaire study. The proportion of patients who completed the scan experience questions was arguably quite low at 54%. However, this is in line with postal survey completion rates observed in other similar studies.^33^ We did consider issuing reminders to patients to increase response rate, but decided against this so as to not increase patient burden at a difficult time: patients had to complete and return two questionnaires within 1 month of a new cancer diagnosis. Although those who took part seem representative of Streamline trial participants overall (judged by our comparisons of registered and recruited patients), our sample may not represent all patients who may undergo WB-MRI in daily clinical practice. Patients in our study were relatively young compared to the typical age of diagnosis with lung or colorectal cancer and it is possible that scan acceptability is greater in younger patients. However, the study was done within the context of a large multi-institution study of WB-MRI, and the results are very likely to representative of most National Health Service institutions. The study was powered to detect clinically meaningful differences in perceptions of burden generated by WB-MRI and standard scans, while the power calculation prior to the start of the study assumed we would be using paired samples *t*-tests rather than Wilcoxon signed-rank tests, significant differences were still detected with the latter. Other studies have used much larger numbers to try and predict poor tolerance of MRI.^8^ It is possible our null findings for some predictors (*e.g.* age, gender and cancer type) and findings of borderline significance for the role of deprivation in adjusted analyses, may be due to lack of statistical power to detect small effects. Patients were asked to complete the baseline questionnaire at the point of trial registration, with the post-staging questionnaire 1 month later. Scan timing meant that at baseline some patients had already completed WB-MRI by the time they completed the baseline questionnaire and a whole month had elapsed before they were asked to answer the post-scan evaluation questions. This may have introduced some recall bias into their responses. However, recalled experience some time after the event may have greater prediction for future health behaviours than immediate recollection.^34^ Some patients may have been aware of their diagnosis at the time of completing the baseline questionnaire, when distress levels were assessed. We did not ask people whether or not they knew their diagnosis at baseline, but rates of distress among people undergoing investigations for suspected cancer are similar to those among people with a confirmed diagnosis, so this is unlikely to have affected the results observed.^1^

It would have been useful to quantify patient comorbidity with scores such as the Charlson score.^35^ However, such scores are time-consuming and collection of complete and clean data were not possible with our resources. As noted in the methods, however, self-report measures of comorbidity have been shown to be valid^17, 18^ and offer a more cost-effective method of data collection than medical record-based measures.

A further limitation is that our study focused on scan experience, and although a number of questions were asked about scan acceptability, recovery time, and satisfaction with information, communication and facilities we did not examine patient views about overall appointment time, or how they viewed the time in the scanner * vs * the time waiting before and after the scan. However of note, satisfaction was very high for all these items, and did not differ between scans.

## Conclusions

In conclusion, patients undergoing staging for lung or colorectal cancer found WB-MRI more burdensome than standard CT and PET-CT, although absolute differences in burden scores were small; most patients found WB-MRI fairly or very acceptable. Our findings demonstrate that patients with medical comorbidities, or with pre-existing high levels of psychological distress, tolerate WB-MRI less well, and may, therefore, benefit from additional support.

## Collaborators

The authors of this paper are part of a wider group that form the Streamline trials investigators and include the following collaborators: A Aboagye, L Agoramoorthy, S Ahmed, A Amadi, G Anand, G Atkin, A Austria, S Ball, F Bazari, R Beable, H Beedham, T Beeston, N Bharwani, G Bhatnagar, A Bhowmik, L Blakeway, D Blunt, P Boavida, D Boisfer, D Breen, J Bridgewater, S Burke, R Butawan, Y Campbell, E Chang, D Chao, S Chukundah, B Collins, C Collins, V Conteh, J Couture, J Crosbie, H Curtis, A Daniel, L Davis, K Desai, M Duggan, S Ellis, C Elton, A Engledow, C Everitt, S Ferdous, A Frow, M Furneaux, N Gibbons, R Glynne-Jones, A Gogbashian, V Goh, S Gourtsoyianni, A Green, Laura Green, Liz Green, A Groves, A Guthrie, E Hadley, A Hameeduddin, G Hanid, S Hans, B Hans, A Higginson, L Honeyfield, H Hughes, J Hughes, L Hurl, E Isaac, M Jackson, A Jalloh, S Janes, R Jannapureddy, A Jayme, A Johnson, E Johnson, P Julka, J Kalasthry, E Karapanagiotou, S Karp, C Kay, J Kellaway, S Khan, D Koh, T Light, P Limbu, S Lock, I L ke, T Loke, A Lowe, N Lucas, S Maheswaran, S Mallett, E Marwood, J McGowan, F Mckirdy, T Mills-Baldock, T Moon, V Morgan, S Morris, S Nasseri, N Navani, P Nichols, C Norman, E Ntala, A Nunes, A Obichere, J O'Donohue, I Olaleye, A Onajobi, T O'Shaughnessy, A Padhani, H Pardoe, W Partridge, U Patel, K Perry, W Piga, D Prezzi, K Prior, S Punwani, J Pyers, H Rafiee, F Rahman, I Rajanpandian, S Ramesh, S Raouf, K Reczko, A Reinhardt, D Robinson, P Russell, K Sargus, E Scurr, K Shahabuddin, A Sharp, B Shepherd, K Shiu, H Sidhu, I Simcock, C Simeon, A Smith, D Smith, D Snell, J Spence, R Srirajaskanthan, V Stachini, S Stegner, J Stirling, N Strickland, K Tarver, J Teague, M Thaha, M Train, S Tulmuntaha, N Tunariu, K van Ree, A Verjee, C Wanstall, S Weir, S Wijeyekoon, J Wilson, S Wilson, T Win, L Woodrow, D Yu.
